# A qualitative exploration of perspectives of physical activity and sedentary behaviour among Indian migrants in Melbourne, Australia: how are they defined and what can we learn?

**DOI:** 10.1186/s12889-021-12099-4

**Published:** 2021-11-13

**Authors:** Siona Fernandes, Cristina M. Caperchione, Lukar E. Thornton, Anna Timperio

**Affiliations:** 1 Physical Activity and Nutrition (IPAN), School of Exercise and Nutrition Sciences, Deakin University, Geelong, Australia; 2 School of Sport, Exercise and Rehabilitation, University of Technology Sydney, Moore Park Precinct, Sydney, NSW Australia

**Keywords:** Indian migrants, Physical activity, Sedentary behaviour, Cultural perspectives, Holistic notion, Qualitative inquiry

## Abstract

**Background:**

Although perceptions of what constitutes physical activity (PA) may vary between culturally diverse populations, very little research has explored the perceptions of PA among Indian migrants. This study aimed to identify how PA and sedentary behaviour (SB) are defined and describe how these definitions are shaped by cultural background and migration among a sample of Indian migrants living in Australia.

**Methods:**

Using an exploratory qualitative approach, semi-structured interviews were conducted with twenty-one Indian migrants living in Melbourne (10 men and 11 women; age range: 18 to 65 years). Interviews were audio-recorded and transcribed verbatim. Data were coded and analysed inductively using thematic analyses.

**Results:**

Data revealed two emerging themes: 1) Holistic perspectives related to PA, where although the majority of participants described PA as “any sort of movement”, or “physical exercise”, several of these descriptions had interwoven ideas related to the mind (mind-body connect), social, cultural, and to the outdoor environment; 2) Broader perspectives for SB, where descriptions of SB as “not having movement”, “doing nothing” or “being lazy”, were shaped with ideas of purpose and duration. Women spoke about how their perspectives of PA and SB may be shaped by native Indian experiences, particularly the gender roles, social caste, and regional subcultural norms which they perceived were important to consider among women who migrate to western settings.

**Conclusions:**

Cultural background is important in shaping the perspectives of PA and SB among Indian migrants in Australia. Practitioners and researchers should consider the varying perspectives of PA to communicate and promote PA among migrant populations more effectively.

**Supplementary Information:**

The online version contains supplementary material available at 10.1186/s12889-021-12099-4.

## Background

Physical inactivity contributes to the global non-communicable disease (NCD) burden, with costs to health care systems attributable to inactivity conservatively estimated at INT$53.8 billion globally and further costs associated with INT$13.7 billion in productivity losses [1, 2]. South Asian descendants namely from India, Maldives, Pakistan, Bhutan, Sri Lanka, Nepal, and Bangladesh are disproportionately liable for NCD-related mortality globally [3]. It is suggested that Asian Indians have a genetic predisposition that perpetuates their NCD-risk at younger ages (< 40 years) [4–6] and present a greater risk of NCDs when compared to western European and other Asian populations [7]. The health risk may be greater for Indian migrants when compared to native counterparts [8], as health-risks may in part be attributed to having lower levels of physical activity and increased sedentary behaviour in western countries, such as the US, UK, Canada, New Zealand and Australia [8–13]. The healthy immigrant effect suggests that although recent migrants who fulfill rigorous health screening requirements upon entry have a greater health advantage than their native counterparts, yet over time their health declines [14]. It is suggested that migrant populations from non-western communities may succumb to poor lifestyle behaviours such as low physical activity and increased sedentary behaviour when adapting to westernised national cultures and infrastructure [15]. This is critical to consider for a genetically predisposed population, and where socio-cultural influences in the process of adapting to a new country (acculturation) can accentuate the health risk, and over time perpetuate the public health challenge [16, 17]. Increasing physical activity among Indian migrants is an important solution in addressing their burden of multiple NCDs, but making efforts to increase physical activity behaviour, and limiting sedentary behaviour necessitate that we understand how such behaviours are defined or conceptualised by Indians in their migrant-setting.

Physical activity is typically defined in the literature as ‘any bodily movement produced by skeletal muscles that result in energy expenditure’ [18 , p.126]. It includes occupational, household, travel, and leisure (e.g., sport, exercise performed for fitness purposes, yardwork) physical activity [19]. The consensus definition of sedentary behaviour is ‘any waking behavior that expends energy less than 1.5 METs while in a sitting or reclining or lying posture’ [20 , p.^5^]. These operational definitions, however, may not reflect how the public views these behaviours. Very few studies have explored how culturally, and linguistically diverse (CALD) migrant populations define or conceptualise physical activity and sedentary behaviour. Qualitative insights revealed that Cambodian, Mexican, Sudanese, and Somalian immigrants and refugee communities living in the United States, had conceptualised their knowledge and practice of physical activity with ideas of purposiveness when engaging in exercise, sport, and household tasks [21]. Indian migrants in the United States and Canada defined physical activity as informal and automated behaviour and considered perspectives related to their spirituality and cultural experiences (yoga, pranayama-breathing) [22, 23]. In Australia, the different cultural and ethnic identities (family vs individual) were regarded as potential explanations for the ‘unplanned’ norm of physical activity among Indian and Sri Lankan migrants compared to the planned exercise norm among Anglo-Australians [24]. The CALD-Physical Activity Mapping Project outlined the array of differing perceptions across Indian, Greek, Vietnamese, Filipino, Samoan, Sudanese, Bosnian, Arabic-speaking, and Spanish communities living in Queensland, Australia [25]. In that study, Arabic and Sudanese women attributed their ideas solely to weight loss while Indian and Vietnamese described perspectives related to family, work, and social settings. Such perceptions of physical activity and sedentary behaviour within CALD populations often differ from the operational definition of physical activity.

Currently how physical activity and sedentary behaviour may be understood among Indian migrants is limited to perspectives from older participants (70 + years) [23], those with a clinical diagnosis [22, 26], or considering a single Indian regional sub-culture (e.g. only Punjabi migrants) [22]. For a growing Indian population in Australia, understanding how Indian migrants perceive physical activity and sedentary behaviour will be important to tailor their health promotion strategies and interventions [25, 27]. Hence, this study aimed to 1) identify how physical activity and sedentary behaviour are defined by Indian migrants living in Australia; and 2) describe how these definitions are shaped by cultural background and migration.

## Methods

This is an exploratory qualitative study that uses thematic analysis to unravel the meaning and perceptions of physical activity and sedentary behaviour among Indian migrants [28]. Such an approach has previously served as a framework for revealing conceptual depth about the thoughts, experiences, and feelings of interest among specific populations [29]. Semi-structured interviews and the use of prompts enabled the researcher to uncover new meaning and in-depth descriptions of any experience of interest within a free-flow, and personalised setting [30]. The study was approved by the Deakin University Human Ethics Advisory Group-Health (HEAG-H 93_2019) on the 4th of July 2019.

### Sample recruitment and settings

All interviews occurred in Melbourne, Australia between August to December 2019. Melbourne currently holds the fastest population growth rate of capital cities in Australia, with overseas-born migration the greatest contributor [31]. Following England, Indians were the second-largest overseas-born resident population in the state of Victoria in 2016 [31].

Purposive sampling was used to ensure a diverse selection of individuals [32]. To be eligible, participants had to have resided in Australia for one year or more, with an intended length of stay of more than two years and be fluent in English. Indian descendants from countries other than India (e.g., Fijian Indians) and those below 18 years and above 70 years of age were not eligible. Those with a reported chronic illness or physical disability that limited their capacity to engage in any physical activity were also not eligible as the interview included questions to explore barriers and enablers of physical activity (not reported here), which may differ substantially for this group. Refugees and new arrivals within their first year in Australia were not included as they may have different experiences concerning these behaviours. Recruitment also sought to include first and second-generation Indian migrants. The criteria for first-generation participants included those born in India, and second-generation included those self-identified as Indians either born or migrated to Australia before the age of 12 with one Indian-born parent. This age cut-off has been used in other studies [33, 34].

Participants were recruited through word-of-mouth, social-media channels (e.g., Facebook), and posters at various community centres, local libraries, local Indian grocery stores, and cultural organisations. Social-media channels and posters included a link to an online survey that directly screened respondents for eligibility. The few respondents who directly contacted the research team member to register their interest in participating were screened for eligibility by telephone. Those who met the eligibility criteria were emailed information about the study. All participants provided written informed consent prior to the start of the interviews.

### Procedures

Additional demographic details, for example, household, occupation, education, and reason for migration (open-ended question) were obtained before each interview. Participants self-selected their preferred interview mode, selecting from skype, telephone, or in-person at a public location. Interviews were conducted in English by the first author (SF) for a duration of 35 to 45 min. Where appropriate, field notes were used to record post-interview contextual details such as interaction styles and emotions. Considering the diverse participant ages, generation, and sub-cultural profiles, achieving thematic saturation was approximated after 17 interviews wherein descriptions of experiences (richness) were considered adequate for analysis (i.e. sufficient knowledge answering the research aim) and within the allotted timeframe [35, 36].

#### Interview guide

The interview guide (Supplementary Table 1, Additional File 1) was informed by the five-phase-systematic methodological framework for semi-structured interviews [37]. It was developed to gather information about the study aims and a broader research question related to influences on physical activity and sedentary behaviour among Indian migrants (not presented here). Participants were first asked questions related to their physical activity, followed by questions about sedentary behaviour. For example, participants were asked “*Describe what physical activity means to you?”* or “*What does the term sedentary behaviour mean to you?*”. Prompting cues were used to draw out further detail and participants were encouraged to provide examples to support their ideas and/or perceptions. Prior to conducting the interviews, the questions were piloted on three participants from the target population to ensure that the interview guide was salient and culturally appropriate. After the interview, each participant received a gift card for their time and contribution.

### Data analysis

Each interview was audio-recorded and transcribed verbatim by an external transcription service. Each transcript was checked for accuracy by the first author (SF) before commencing the data coding process. A thematic inductive coding approach was employed using the Braun & Clarke framework [28]. A codebook, containing codes and a hierarchical structure of overarching codes and sub-codes, was developed, and checked iteratively by the two authors (SF, CC). Overall, coding a single undivided passage of the data was the preferred unit of analysis [38]. Line-by-line coding was also used to identify certain elements within a single unit that added depth to the overall meaning [39]. Such an approach enabled a closer view when unpacking novel and important aspects while keeping the broader focus of the overall aims and considering the overall responses of the participant as a single unit [38]. The first author presented the themes and sub-themes separately for physical activity and sedentary behaviour, to all co-authors (CC, AT, LT) who cross-checked, refined, and finalised the themes. The authors discussed the coding process making necessary revisions to the coding framework, addressing general clarifications, and ensuring safety standards of data management were maintained. The first author conducted all the interviews and the data analyses. The first author shared similar cultural background as the participants and has previously worked with Indian migrants, including the conduct and tailoring of physical activity research for Indian migrants. As a cultural insider, the first author engaged in reflexive practices (e.g., journaling) to ensure rigour and trustworthiness were maintained [40]. The co-authors, were cultural outsiders to the research process who carried the necessary knowledge and expertise to conduct research in physical activity and sedentary behavior; one co-author has also previously worked with migrant communities. The data and coding were managed with Nvivo Software for Mac, Version 12 [41]. A sample of the final coding framework is attached separately (Supplementary Table 2, Additional File 2).

## Results

Of 44 individuals who responded, 32 were eligible to participate and were emailed further information about the study and consent forms. Of these, 21 provided written informed consent and completed an interview. Nine interviews were conducted in-person, eight via skype, and four over the telephone. First-generation participants (*n* = 18) were originally from diverse regional sub-cultural backgrounds of India: two participants each from Punjab, Maharashtra, Karnataka, Tamil Nadu, Goa, and one participant each from Andra-Pradesh, Delhi, Kerala, Jharkhand, Uttar-Pradesh, Kolkatta, Orissa, Rajasthan, Madhya-Pradesh, Jammu, and Haryana. For second-generation (*n* = 3), one participant each had self-reported their origins from Kolkatta, Karnataka, and Maharashtra. Reason for migration mainly included seeking education and job opportunities (*n* = 8), followed by relocation, or reuniting with their family members (*n* = 5), improved quality of life (*n* = 3), and to gain overseas experience (*n* = 3). Additional participant characteristics are presented in Table 1.

**Table 1:** Participant characteristics

	Total sample (*N* = 21) N (%)
**Gender**	
Men	10 (47%)
Women	11 (53%)
**Age** (years)	
18–35	14 (66%)
36–55	6 (29%)
56–65	1 (5%)
**Education qualifications**	
University/tertiary degree	21 (100%)
**Generation**	
First-generation	18 (86%)
Second-generation	3 (14%)
**Migration information**	
*Year’s resident in Australia*	
1 < 3	6 (29%)
3–10	7 (33%)
> 10**	8 (38%)
*Age at migration (years)*	
< 18 **	4 (19%)
19–25	9 (42.9%)
26–35	7 (33%)
36–45	1 (5%)
**Current work**	
*Total hours at work per week*	
< 20	2 (9.5%)
20–40	14 (67%)
> 40	5 (24%)
**Household characteristics**	
*Additional household occupants*	
Live alone	3 (14%)
1–2	5 (24%)
3–5	9 (42.9%)
> 6	3 (14%)
*Children in the household*	
0	15 (71.5%)
1–3	6 (28.5%)
*Number of drivable motor vehicles*	
0	5 (24%)
1	8 (38%)
> 2	8 (38%)

**Second-generation those migrated from India to Australia before 12 years of age with one Indian-born parent

The qualitative results revealed two overarching themes: 1) The holistic perspective for physical activity sub-themes of which related to mind-body, social, cultural, and environmental perspectives; 2) Broad perspectives of sedentary behaviour related to ideas of purpose and culture which they weaved into the perceived benefits and consequences of sedentary behaviour. Given the disproportionate number of second-generation to first-generation participants, no distinctions by generation were made within these findings.

### Perspectives of movement and intensity

The majority of participants described physical activity as “any sort of movement”, or “physical exercise” mainly performed for leisure (gym, sport), or active travel (walking, cycling), occupational (shelving), and housework practices (cleaning, cooking, gardening). Some considered not engaging in sedentary behaviour as physical activity. As one man stated, “*The term physical activity to me means working out, doing physical exercise or walking or jogging or whatever. Just not being sedentary is my definition of physical activity*”. Similarly, a woman offered, *“It [physical activity] means moving a lot. Doing things, not sitting”*. Ideas related to intensity and energy expenditure emerged when few participants perceived planned exercise behaviours (gym, sport) required greater physical effort over lower intensity activities such as walking. As one man summarised, *“I like to push myself physically to the limit where my body starts to shut down and almost get exhausted, that to me is exercise, rather than just going for a nice, gentle walk”*.

#### Holistic perspectives

For some participants, physical activity was not only about engaging in bodily movement but a way of engaging the mind. As one man stated, “*Physical activity means the overall development of a person … mentally as well as physically”*. Such descriptions are summarised by two other participants weaving in this mental perspective in the context of recreational sport and traditional yoga.

*“For me, it’s getting your mind off the regular stuff you do, and it’s not just running, it’s getting engaged in some kind of an activity where you’re not thinking about work or regular day-to-day stuff. For example, tennis is one thing. You’re also thinking about the ball. When you’re playing cricket, you’re thinking about the timing. So, giving your mind a break from the regular day-to-day tasks and activity, that’s physical activity for me”. [Male participant].*

*“I realise physical activity doesn’t only mean you move your body, you should be aware of every part even when you’re exercising, doing yoga, or running … For me, physical activity means awareness to my body, whatever I’m doing”. [Female participant].*

#### Social, cultural, and environmental perspectives

Although women mainly pointed to ideas about physical activity in connection with people and nature (i.e., outdoor environment), one man spoke of his connection of physical activity to nature when stating, *“I don’t know, it [trail running] just has a calming feeling when you’re running outdoor, with more trees”*. When defining physical activity, one woman stated, *“Like it means working out, working up a sweat. Sometimes physical activity is also being outdoors and walking with friends”*. Similarly, reflecting on her traditional Indian experiences where agriculture was considered the main source of physical activity another woman stated, *“So life changed after moving abroad I think in terms of physical activity. Physical activity for me, [is] being close to nature and then doing something. So that really changed”*. Another woman also emphasised the socio-environmental connection in view of increasing physically active behaviours and reducing sedentary practices: *“I think if you want physical activity, you enjoy it with more people, with more friends, with more nature. I think it motivates you to become less sedentary”*. Ideas of social connection also emerged when speaking about traditional/cultural modes of activity. For instance, one woman shared how traditional festivals that involved cultural dancing perpetuated the social connections with members of their community/culture. Connecting with their community members while engaging in physical activity practices was considered an integral part of the Indian culture.

*“I think dance is also part of physical activity because it doesn’t only make you fit, it makes you happy as well. Overall, all Indians love to dance. I think that’s one part of our culture which is amazing. It [dance] makes people healthy, it makes people engage with others, the social contact which nowadays people are lacking, [is still] there in our culture. When I go back, we have certain festivals where you gather around, like harvesting festival, and dance”. [Female participant].*

A small portion of women spoke of how perspectives of physical activity can be shaped by their social-cultural norms and practices. Their descriptions revealed that an Indian experience may not be homogenous but a conglomeration of diverse norms and practices arising from distinctions of class, caste, and regional sub-cultural norms and practices. Describing how nuances of class or caste intersections of Indian culture may shape the gender-based experiences of physical activity these women offered,

*“… You come from a certain class or a certain caste, where women are expected to do traditional roles. So, a mother is just a mother, and her role is defined. She has got to cook she has got to look after the family. She’s not somebody who is encouraged to take up any hobbies”. [Female participant].*

Her quote highlights the notion that the social stratifications of class and caste inherent to the Indian culture can position native Indian women to adhere to performing their traditional gender roles. She also pointed to how perspectives and preferences of physical activity for women may be shaped by sub-cultural norms that were important to consider when these women migrate to western settings. She recognised that culturally shaped perspectives and practices of physical activity may not be consistent with perspectives and practices around physical activity in their migrant/western contexts.

*“With one demography is not the same, from up north to down south. It’s [a] different culture, different upbringing. In south India, every family household encourages girls to take part in dance, they should learn [dance], even if they won’t take it up as a profession, at least as a hobby they could do it. But that may not be the case in another part of India … but when you come to Australia, you bring those things with you which may not be applicable here”. [Female participant].*

Similarly, another woman supported the idea that experiences of physical activity may differ for individuals across the Indian demographic. For her, activities traditionally performed for daily living in her regional subculture shaped her understanding that physical activity was an informal practice, rather than a planned exercise behaviour. Her quote suggests that among men and women in rural Indian agricultural communities, physical work as part of daily living may be viewed as an important and legitimate form of physical activity that creates little distinction along gender lines.

*“For every individual or every community, every society, physical activity has different meanings. I’m from …. a state mainly famous for agriculture … In my state people give a lot of emphasis on physical activity. So, there was nothing like a cultural difference for men and women. So, for me, doing some sort of physical work comes in the [form of] physical activity even if you’re not going to the gym”. [Female participant].*

#### Perspectives of intent or purpose on sedentary behaviour

A small number of participants expressed that they did not understand the meaning of the term sedentary behaviour. However, for most participants, sedentary behaviour conveyed a state of idleness described as “just sitting”, “doing nothing”, “being lazy” or “not moving” when sitting and lying. Several participants had interwoven other ideas related to the purpose (underlying reason) and duration (length of time). Narrating how the idea of purpose is tied to shaping the meaning and practice of sedentary behaviour, one man stated, “*Like you are just sitting down for nothing, or you are sitting down and doing something creative. It all depends on what you are doing … Being creative or just lying or being lazy all the time?*”. Similar ideas of purpose and duration were shared by other participants when stating,

*“When I scroll down Facebook, for me it’s something very important because I just read the news. So, I never included it as a sedentary lifestyle, because to know about the news and politics, I’m very interested. That’s why I didn’t tell you I scroll down Facebook, and I don’t add it to my sedentary life”. [Female participant].*

*“It depends on if it goes on for a while and it starts impacting your health, that’s what I believe is sedentary. Whereas having ‘me time’ sitting, watching TV, having time off once now and then actually is helpful. As long as you’re not lying down on the couch for the whole day”. [Male participant].*

When exploring past cultural experiences, two participants highlighted how activities of daily living were performed adopting a sedentary ‘position’ in India. Their descriptions suggested that in India it was customary for them to engage in activities of daily life in a floor-seated or squat ‘position’. Such cultural experiences of, or reflections on, sitting or squatting to perform important activities of daily living may in some part underpin views on intent and purpose shaping what is and isn’t considered sedentary behaviour.

*“Most Asian communities, they sit in a certain pattern [squat]. In terms of cooking, our toilets and everything was different, which now I think [is] approved by the scientists and researchers, why it was important to sit in certain behaviours...These kinds of sedentary behaviours really affect when we are coming from [a] certain environment and then jumping to here [Australia]”. [Female participant].*

*“We used to sit a lot more on the floors, back in India, than on the couches, even though we had couches. I definitely think it’s one of those cultural things where people prefer just sitting down on the floor a lot more. When I was young, we always used to sit on the floor to eat, to do everything. To do our homework, to study”. [Male participant].*

In defining sedentary behaviour, the participants’ ideas of intent and purpose also surfaced in discussions of the perceived benefits and consequences of being sedentary, particularly when sitting and lying. For instance, there was a perception that sitting and lying benefits physical and mental relaxation, helps connect with friends and family members, however while sitting with the intent of “doing nothing” or being "lazy" was perceived as having negative health consequences, such as physical (back/join) discomfort, weight gain, negative mood, and social disconnection. As one woman stated, “*I would put on weight, I would have low moods, I would be not as social as I want to do and not being social and not having friends would create other problems”*. Narrating how the idea/purpose of sitting is essential as it provides holistic health benefits from physical, mental to the social contexts, they offered,

*“[sitting] helps you relieve and relax, and come into your good mood, because of stress you had in the job. So, it [sitting] helps you get back to normal”. [Male participant].*

*“It’s kind of a bonding time I have with my son. Sitting down is an important part of my life when you have a kid and it should be in anybody’s life, I feel... sitting at home with your family, your friends or with my son, I don’t think it has any negative impact”. [Female participant].*

## Discussion

Among a sample of Indian migrants living in Melbourne, Australia, this study explored how physical activity and sedentary behaviour were defined, further describing how such perceptions were shaped by culture and migration. When defining physical activity, Indian migrants had interwoven ideas of bodily movement with broader ideas related to mental engagement (mind-body), social, cultural, and environmental aspects. For sedentary behaviour, they shared ideas related to purpose and duration when conveying the lack of movement and energy expenditure.

### Broader perspectives

The findings agree with previous qualitative studies that found broad conceptualisations of physical activity (e.g., purposiveness, passivity, cultural, familial, social) among Indian migrants, other immigrants, refugees, and native communities at risk of non-communicable diseases [21–24, 27, 42]. The conceptualisation of physical activity among non-Indian immigrants and refugees has been attributed more to the social, economic, and environmental factors they experienced in developed countries and less to their cultural norms [21]. However, qualitative insights from studies targeting Indian migrants revealed that Indian migrants experienced and perceived physical activity with ideas related to culture (yoga), spirituality (Hinduism), familial, social, and outdoor environmental experiences, which parallel the broad ideas expressed by participants in our study [22–24, 26, 43]. Such broad perspectives may also stem from Indigenous Indian religion and spiritual traditions and philosophies. Authors in the discipline of Indian psychology emphasised that in addition to dependence, materialism, and collectivism, the established set of attitudes/perceptions of an Indian mindset also involved *holistic* aspects that may vary by the diverse ethnic norms within their society [44]. Accordingly, attitudes of knowledge, realities, and existence (i.e. how Indians may think, feel, act) may be distinctive, integrated by diverse aspects such as their physical, social and spiritual experiences [44, 45]. Awareness of such cultural/contextual knowledge (perceptions, attitudes, intentions) as often overlooked in physical activity literature, may be useful when tailoring health promotion messages and population-specific physical activity interventions.

### Culture & Indian experiences

#### Gender perspectives

This study expands the insight on the cultural norms and practices that may shape perspectives and experiences of physical activity for an Indian migrant population. The views expressed by women in this study are supported by several studies that previously accounted for the gender-defined roles of Indian migrant women with low levels of physical activity from adhering to their cultural norms and responsibilities [43, 46–49]. While partaking in physical activity during childhood may be culturally encouraged, meeting cultural expectations during adulthood may necessitate that the Indian woman prioritise her time and responsibilities attending to family needs over engaging in physical activity [43]. Such culturally defined roles may be important to consider when planning physical activity initiatives to engage Indian women, particularly those migrating during adulthood. Previous authors attributed such culturally defined roles to the lower sedentary times (sitting, standing) among South Asian women compared to European women, thus implying that different ethnicities or cultural norms may impact physical activity and sedentary practices [50].

#### Heterogenous perspectives

The participants reiterated what several authors have previously emphasised, that the Indian population is not homogenous but an amalgamation of sub-cultural norms, values, and practices, including distinctions of caste, class, language, and religion [51–53]. For instance, Punjabi Indian migrants in the United States attributed their native agricultural/farming experiences to perceive physical activity as an informal activity of daily living [22]. Similar insights on physical activity as an automated experience, drew from how activities of daily living were performed in the Indian culture, and not attributed as a behaviour that is planned, as the concept of ‘exercise’ in western cultures [24]. Such insights may imply the possibility that the Indian experience may be shaped with distinctive ideas and experiences that may impact an individual’s or group’s preferences of physical activity which is an important issue for future research involving Indian migrant communities. Such insights can validate our findings, expand, and support existing knowledge that may help inform the tailoring of physical activity interventions for Indian migrants globally.

#### Reshaping the conventional lens of sedentary ‘position’

There is limited literature on perceptions of sedentary behaviour among Indian migrant populations. South African Langa community members that conceptualised inactivity with ideas of ‘passivity’ beyond solely adopting the ‘sitting’ position may support our findings [42]. Two participants revealed how traditionally, in India, floor-sitting, and even squatting-positions were being adopted to perform activities of daily life. These findings may be supported by research in other disciplines (e.g. occupational therapy), that identified floor sitting and squatting positions were commonly adopted by Indian [54, 55], and other non-western cultures [56] when undertaking activities of daily living, and recognise culture plays a significant role in how activities of daily living are performed, particularly among ethnic minorities [57, 58]. Some have emphasised the need for awareness of such cultural distinctions in behaviour, not only between different countries but also within each national culture, as such practices may vary between the urban-affluent and rural-lower economic dwellers [56, 59]. Hewes (1957) ‘anthropology of posture’, approximated 1000 types of positions adopted across different cultures when performing such activities [57]. It is plausible, therefore, that the operational definitions around physical activity and sedentary behaviour built solely on energy expenditure (sitting/reclining) position and bodily movement [18, 20] may not adequately reflect the array of such behaviours across different cultures, and possibly within each culture.

#### Perspectives of purpose and duration

Studying adults in the UK, previous authors note the importance of considering the ‘purpose’ underlying sedentary behaviour over the level (time, bouts) of sedentary practices [60, 61]. For instance, perceiving poor health consequences as related to engaging in mindless/passive sitting with prolonged duration, while sitting to socialise with friends and family, or solving puzzles or relaxation were perceived as a beneficial use of sedentary behaviour [60, 61]. Sedentary practices over longer durations were more likely to occur for leisure purposes such as social contexts [61]. While these studies focus on older adults in the general population, their insights support the participants’ perspectives on purpose, duration, and perceived benefits of sedentary behaviour. Such perspectives may be important for a collectivist Indian culture, where social connections with friends, family, and community members form part of their identity and may result in greater sedentary practices in the social/leisure domain than others. More research exploring the contextual/domain-specific experiences of sedentary behaviour of Indian migrants may help identify determinants of their sedentary behaviour.

### Holistic perspectives and culturally relevant insights

Overall, the broad ideas in this study support a need for holistic approaches that incorporate physical, mental, social, environmental, and cultural perspectives when researching, communicating, and promoting physical activity among Indian migrants. It may be that to understand their physical activity and sedentary behaviour we should also consider adjustments of their cultural norms, values, and gender roles with migration. This is particularly important when acculturating to a new culture, where migrants are required to adjust/modify their native cultural values and practices to align with the contextual and cultural values and practices of a western setting/culture [62]. For instance, Indian women moving away from their extended family or rural agricultural backgrounds may rethink the broader perspective such as social connection to physical activity from connecting with their community and extended family members, and with lack of social support in their migrant settings, may alter their perspective to consider physical activity as a planned exercise, such as going to the gym. Similarly, they may consider sitting practices important to help build their social connections as migrants. Culturally tailored interventions that have incorporated such holistic approaches have led to improved health outcomes, including physical activity practices among ethnic minority women in Canada [63]. Holistic perspectives are particularly important for other ethnic/indigenous communities in Australia and South America, where identities of self and health-related practices are also shaped with ideas of mind-body, social, landscape, psychological, ideas of sharing, familial and communal harmony, and balance of the external world and inner-self [64, 65]. Giving importance to understand the impact of migration on re-shaping such indigenous/native expressions of physical activity and sedentary behaviour in addition to cultural and social influences would complement efforts to support their participation in physical activity.

### Limitations

This study solely considered the views of participants fluent in English and did not include any participants older than 65 years. While we sought to include second-generation Indian participants, only three were recruited and no comparisons could be made, limiting the transferability of the findings. Participants were provided with the choice to participate in the interview in-person, by telephone, or online to minimise participant burden. However, these different modes of data collection may have affected responses.

## Conclusions

This study provides a rich and nuanced account of how physical activity and sedentary behaviour are understood and defined among an Indian migrant population. Framing physical activity with a holistic lens and using holistic approaches to promote physical activity are thus encouraged. Cultural behaviours/practices can have implications for how we consider, categorise, and measure sedentary behaviour, diverging from the one size fits all approach. Such insights can support efforts to tailor interventions and effectively communicate policies on physical activity for the Indian migrant population.

## Supplementary Information

**Additional file 1: Table S1.** Interview guide. This table displays the lead interview questions and potential prompts developed for physical activity and sedentary behaviour

**Additional file 2: Table S2.** Sample coding framework. This table provides a sample of the nodes, category descriptions and codes of the final coding framework

## Data Availability

The dataset analysed for the current study are not publicly available due to ethical restrictions related to the consent given by participants at the time of study commencement. An ethically compliant dataset may be made available by the corresponding author on reasonable request and upon approval by the Deakin University Human Research Ethics Committee.

## Abbreviations

CALD: Culturally and linguistically diverse
PA: Physical activity (abstract only)
SB: Sedentary behaviour (abstract only)
