# Supplementary material for: A qualitative exploration of perspectives of physical activity and sedentary behaviour among Indian migrants in Melbourne, Australia: how are they defined and what can we learn?
Source: BMC Public Health. 2021 Nov 13;21:2085. doi: 10.1186/s12889-021-12099-4 (PMC8590373; doi:10.1186/s12889-021-12099-4)
Supplement: Supplementary file 1 — Additional file 1: Table S1. Interview guide. This table displays the lead interview questions and potential prompts developed for physical activity and sedentary behaviour [file 12889_2021_12099_MOESM1_ESM.pdf]

Additional files

Additional file 1: Table S1. Interview guide.
This table displays the lead interview questions and potential prompts developed for physical activity and sedentary behaviour.

Interview route	
Physical activity	Sedentary behaviour
▪ Describe what physical activity means to you?<br>▪ Is staying active more or less important to you?<br>▪ Can you give me some examples of type of activities you experience in a typical day?<br>▪ Context of physical activity: When? Where? With whom?<br>▪ At present what helps you to engage in physical activity? (facilitators - prompt)<br>▪ What challenges you to engage in physical activity? (barriers - prompt)	▪ Describe what the term sedentary behaviour means to you?<br>▪ Can you give me some examples of sedentary activities you experience in a typical day?<br>▪ Context of sedentary activities: When? Where? With whom?<br>▪ Describe benefits to engaging in such sedentary activity (E.g., sitting, lying/sleeping)?<br>▪ Describe negative effects/limitations of engaging in such sedentary activities?<br>▪ How has current work-life impacted the level of sedentary activity? (facilitators/barriers - prompt)
Prompts as necessary	
Psychosocial: Perceptions, values, attitudes, definitions	
Institutional: Occupational setting, workplace facilities	
Social & cultural: Social support, Indian /non-Indian community, traditional/cultural modes/practices	
Social & material resources: Area deprivation, opportunities in life	
Physical environment & opportunity: Neighbourhood, parks, open spaces, safety, facilities	
Migration: Acculturation, equal opportunities, past-present comparisons	
Health & health communication: Health information, overall health	
