# Supplementary material for: A qualitative exploration of perspectives of physical activity and sedentary behaviour among Indian migrants in Melbourne, Australia: how are they defined and what can we learn?
Source: BMC Public Health. 2021 Nov 13;21:2085. doi: 10.1186/s12889-021-12099-4 (PMC8590373; doi:10.1186/s12889-021-12099-4)
Supplement: Supplementary file 2 — Additional file 2: Table S2. Sample coding framework. This table provides a sample of the nodes, category descriptions and codes of the final coding framework [file 12889_2021_12099_MOESM2_ESM.pdf]

Additional files

Additional file 2: Table S2. Sample coding framework.
This table provides a sample of nodes, category descriptions and codes of the final coding framework.

Top level Nodes	Category *[Description]*	Codes
**Physical activity perspectives** ***[Ways of defining]***	Importance<br>*[Reasons physical activity is important]*	Image - aesthetics<br>Improved fitness/function<br>Mental<br>Mental & physical/body weight
	Perceived benefits<br>*[Perceptions of the benefits of PA]*	Bodyweight (aesthetic):<br>Related to body image [men]<br>Related to body image [women]<br>Energy and function<br>Mental - Mood<br>Physical with mental context<br>Social<br>Other positive health outcomes
	Meaning and definition<br>*[Various aspects related to understanding of physical activity]*	Being active, moving, not sitting<br>Other aspects:<br>Cultural<br>Connection with mind and body<br>Involves people (social) and outdoor<br>Unconscious over planned
**Sedentary behaviour -perspectives**	Perceived benefits<br>*[Participants views on the benefits of sedentary practices]*	Family time<br>No benefit<br>Reading learning<br>Rest relax recover

		Socialize
		Work & hobbies
		Time for self
	Perceived negative consequence	Back & joint, stiffness
		Brain function & focus
		Get lazy
		Health conditions
		Less social
		Mood & negative thoughts
		None with physical activity
		Weight gain
	Broader meaning	Don’t know
	[definitions and understanding of sedentary behaviour]	Not moving, doing nothing, lazy
		Other-
		Balance with other activities
		Cultural - past observations of women’s sitting
		Cultural - squat and floor sitting
	Overlaps with perceived benefits and consequences	Ideas of duration
		Ideas on energy expenditure
		Ideas on posture
